# Anodal Transcranial Direct Current Stimulation as an Adjunct to Physiotherapy in Lacunar Stroke

**DOI:** 10.7759/cureus.104359

**Published:** 2026-02-27

**Authors:** Ayesha Juhi, Shreya Sharma, Dinesh Bhatia, Suman Dhaka, Rajesh Kumar, Deepak Kumar, Pritam Choudhary, Pradosh Kumar Sarangi, Himel Mondal

**Affiliations:** 1 Department of Physiology, All India Institute of Medical Sciences, Deoghar, Deoghar, IND; 2 Department of Biomedical Engineering, North-Eastern Hill University, Shillong, IND; 3 School of Liberal Arts and Center for Brain Science and Application, Indian Institute of Technology, Jodhpur, Jodhpur, IND; 4 Department of Internal Medicine, All India Institute of Medical Sciences, Deoghar, Deoghar, IND; 5 Department of Physical Medicine and Rehabilitation, All India Institute of Medical Sciences, Deoghar, Deoghar, IND; 6 Department of Radiodiagnosis, All India Institute of Medical Sciences, Deoghar, Deoghar, IND

**Keywords:** cognitive recovery, fugl-meyer assessment, lacunar stroke, montreal cognitive assessment, motor recovery, neuromodulation, physiotherapy, stroke rehabilitation, tdcs, transcranial direct current stimulation

## Abstract

Lacunar strokes affecting deep subcortical motor pathways often lead to significant disability despite their small lesion size, and recovery can remain incomplete with physiotherapy alone. Transcranial direct current stimulation (tDCS) has emerged as an accessible neuromodulation tool that may enhance post-stroke rehabilitation, though evidence from resource-limited settings in India is sparse. This case report describes a 56-year-old man with hypertension and type 2 diabetes mellitus who developed acute left-sided weakness, imbalance, and mild facial asymmetry, with CT imaging confirming a right-sided lacunar infarct involving the basal ganglia and corona radiata. At admission for rehabilitation at All India Institute of Medical Sciences (AIIMS), Deoghar, Jharkhand, India, he presented with moderate to severe motor impairment (Fugl-Meyer Assessment (FMA) score: upper extremity (UE) 21/66, lower extremity (LE) 11/34) and mild cognitive deficits (Montreal Cognitive Assessment (MoCA) score: 16/30). He underwent a six-week program that integrated anodal tDCS (2 mA for 20 minutes, five days per week), delivered over the ipsilesional primary motor cortex using a Neurostim device (Neurosoft LLC, Ivanovi, Russia), combined with physical physiotherapy. By week 4, he demonstrated improved proximal limb control and sitting balance; by week 6, he achieved supported standing, initiated assisted steps, and showed meaningful improvements in hand function. Post-intervention scores increased to UE 34 and LE 20 on the FMA score and 20 on the MoCA score. At the three-month follow-up, these motor gains were sustained, while cognitive scores remained stable. No adverse effects were reported. This case highlights the feasibility, safety, and potential clinical value of combining tDCS with physiotherapy to support motor recovery and cognitive stability in lacunar stroke, particularly in rural tertiary-care environments with limited rehabilitation resources. Larger controlled studies are needed to refine stimulation parameters, identify responders, and strengthen the evidence base for tDCS in small-vessel ischemic stroke.

## Introduction

Stroke continues to be a primary cause of long-term disability globally, resulting in considerable functional and cognitive impairments among survivors [1]. In India, the growing burden of stroke, particularly in low-resource and rural populations, has drawn attention to the need for affordable and accessible rehabilitation strategies [2]. Lacunar infarcts are small deep-brain lesions affecting regions such as the basal ganglia, thalamus, and internal capsule, caused by occlusion of small penetrating arteries [3]. The small size of these infarcts leads to major motor and cognitive impairments because they damage essential neural pathways between the cortex and subcortical areas [4].

Conventional post-stroke rehabilitation emphasizes physiotherapy and task-oriented exercises aimed at enhancing neuroplasticity via repetitive motor training. Recovery may be prolonged and not fully achieved, particularly in individuals with subcortical strokes [5]. In recent years, non-invasive brain stimulation (NIBS) methods such as transcranial direct current stimulation (tDCS) have emerged as promising adjuncts to traditional rehabilitation [6]. The tDCS delivers weak, constant electrical currents to the scalp to modulate cortical excitability and enhance synaptic efficiency, thereby facilitating functional reorganization in affected neural networks [7,8].

Evidence suggests that anodal tDCS over the ipsilesional primary motor cortex (M1) can enhance motor recovery, while bilateral or dorsolateral prefrontal stimulation may improve attention and working memory [8]. Despite encouraging results from controlled trials, data on its clinical application in resource-limited Indian settings remain sparse. This case report presents the course and outcomes of a patient with ischemic lacunar stroke who received anodal tDCS combined with conventional physiotherapy, demonstrating the feasibility and gradual improvement achievable through neuromodulation-assisted rehabilitation in a tertiary-care setting.

## Case presentation

The patient, a 56-year-old man from Deoghar, Jharkhand, India, was previously independent and active in his daily life. He lived with his wife and children in a semi-urban neighbourhood and worked as a small-scale vendor. He had a known history of hypertension and type 2 diabetes mellitus for the past 15 years, both conditions were under routine medical management with good treatment adherence, and he routinely took telmisartan 20 mg once daily for hypertension and a combination of metformin (1000 mg) and teneligliptin (20 mg) for diabetes mellitus. There was no prior history of stroke, seizures, or cardiac illness.

**Figure 1:**
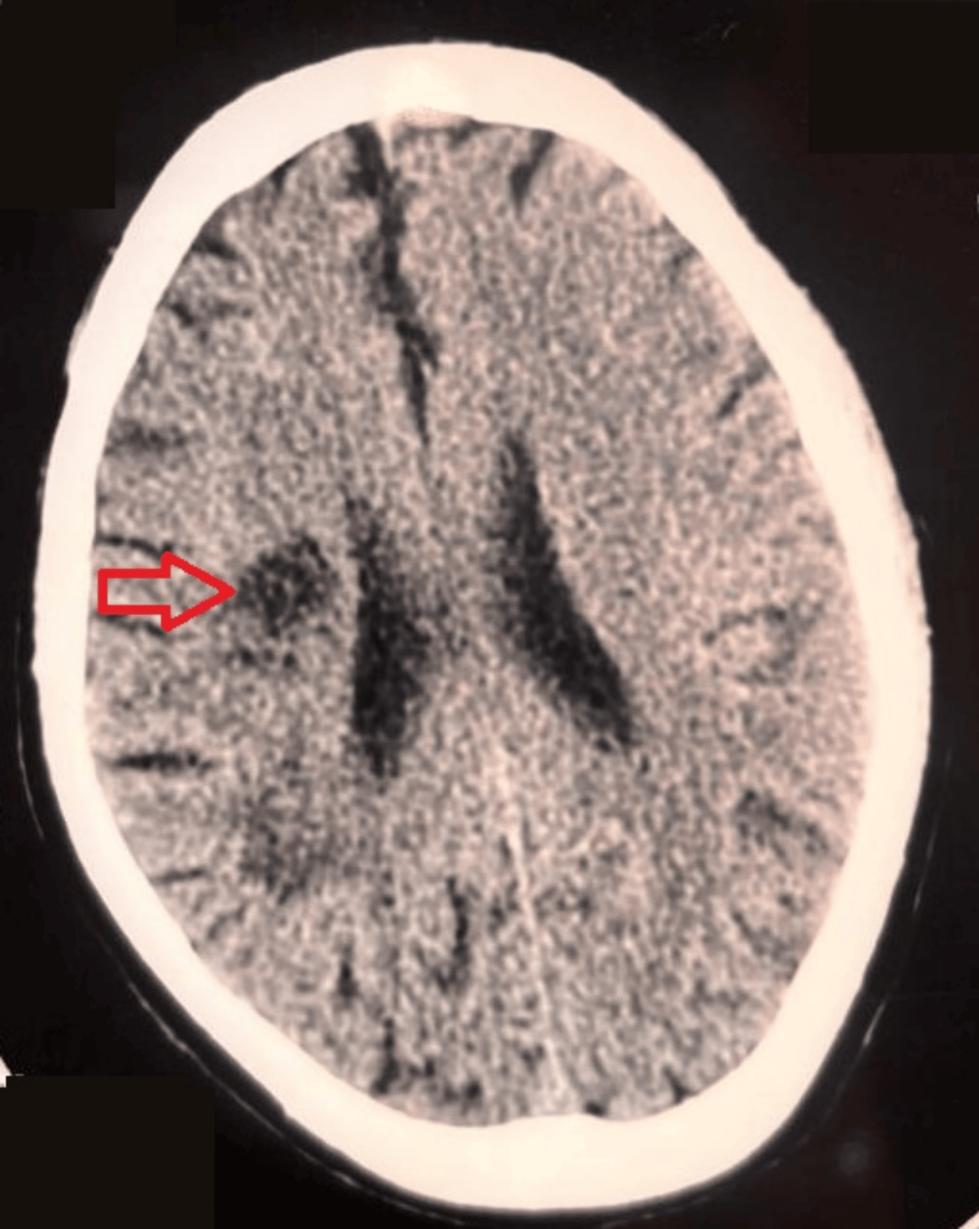
Non-contrast CT scan of the brain showing a lacunar infarct (arrow)

In May 2025, he experienced a sudden onset of weakness and heaviness in his left arm and leg, accompanied by mild facial asymmetry and imbalance while walking. His family immediately took him to a nearby diagnostic center, where a non-contrast CT scan of the brain revealed low-attenuation areas in the right corona radiata and a lacunar infarct (Figure 1) in the right basal ganglia region, consistent with an ischemic stroke. There were no signs of hemorrhage or midline shift.

The timeline of clinical events, assessments, and interventions is summarized in Table 1.

**Table 1:** Timeline of clinical events, assessments, and interventions tDCS: transcranial direct current stimulation, AIIMS: All India Institute of Medical Sciences, FMA: Fugl-Meyer Assessment [9], MoCA: Montreal Cognitive Assessment [10]

Time	Event
May 2025	Stroke onset – sudden weakness in the left upper and lower limbs.
June 2025	The patient was brought to AIIMS Deoghar for continued rehabilitation due to persistent motor and cognitive deficits.
June 2025	Baseline pre-intervention assessment (Motor and Cognitive: FMA and MoCA)
June 2025	tDCS intervention initiated (Anodal tDCS, 2 mA, 20 minutes/session, 5 days/week).
June 2025	Mid-intervention assessment (Week 4) to evaluate progress in motor and cognitive domains.
July 2025	Completion of tDCS intervention (Week 6) - total of 30 sessions combined with physiotherapy.
July 2025	Post-intervention assessment (Motor and Cognitive).
October 2025	Three-month follow-up showing sustained improvements after intervention.

Following acute medical stabilization, he was referred to AIIMS Deoghar for comprehensive rehabilitation. At the time of admission, he arrived in a wheelchair with pronounced left-sided hemiparesis and required assistance for basic mobility. His speech was slurred but comprehensible. He demonstrated psychomotor slowing with mild attentional difficulties.

Baseline motor and cognitive assessments were conducted using the Fugl-Meyer Assessment (FMA) [9] and the Montreal Cognitive Assessment (MoCA) [10]. At this stage, the patient scored 21 out of 66 for the upper limb and 11 out of 34 for the lower limb on the FMA, indicating moderate to severe motor impairment. His MoCA score was 16 out of 30, reflecting mild cognitive impairment, observed in attention, language, delayed recall, and orientation domains (Table 2).

Detailed domain-wise results across all assessment stages are presented in Table 2.

**Table 2:** Domain-wise results across all assessment stages UE: upper extremity; LE: lower extremity; FMA: Fugl-Meyer Assessment [9]; MoCA: Montreal Cognitive Assessment [10]

Assessment Tool	Domain	Baseline: 2 June 2025	Mid-intervention: 30 June 2025	Post-intervention: 15 July 2025	3-month follow-up: 14 October 2025
Motor Function (FMA)	Shoulder/Elbow/Forearm (UE maximum score 36)	14	18	20	22
	Wrist (UE maximum score 10)	4	6	7	8
	Hand/Grasp (UE maximum score 14)	3	3	4	5
	Coordination/Speed (UE maximum score 6)	0	2	3	3
	Hip/Knee/Ankle (maximum score 28)	10	14	16	18
	Coordination/Speed (LE maximum score 6)	1	3	4	4
Total UE (maximum score 66)		21	29	34	38
Total LE (maximum score 34)		11	17	20	22
Cognitive Function (MoCA)	Visuospatial / Executive (maximum score 5)	3	3	3	3
	Naming (maximum score 3)	2	2	2	2
	Attention (maximum score 6)	3	3	4	4
	Language (maximum score 3)	1	2	2	2
	Abstraction (maximum score 2)	1	1	1	1
	Delayed Recall (maximum score 5)	2	3	3	3
	Orientation (maximum score 6)	4	5	5	5
Total MoCA (maximum score 30)		16	19	20	20

The tDCS intervention was delivered using a Neurostim device (Neurosoft LLC, Ivanovo, Russia), a clinically certified neuromodulation system widely used in stroke rehabilitation research. Stimulation was administered with a conventional bipolar montage, placing the anodal electrode over the ipsilesional primary motor cortex M1 to enhance cortical excitability, while the cathodal electrode was positioned over the contralateral supraorbital region (Fp1) to complete the circuit and minimize unintended motor-area inhibition. Each session consisted of 2 mA constant current for 20 minutes, with a 50-second ramp-up and 50-second ramp-down, parameters selected to maximize patient comfort and reduce abrupt sensory perception during current transitions [11]. These stimulation parameters and montage were chosen based on safety guidelines and the feasibility of using tDCS in a resource-limited rehabilitation environment, as they require less technical infrastructure compared to repetitive transcranial magnetic stimulation (rTMS) while enabling reliable modulation of motor cortex excitability [8].

Each stimulation session was followed by task-oriented physiotherapy emphasizing active-assisted exercises, range-of-motion activities, and gait retraining. Following each tDCS session, the patient received 30 minutes of supervised task-oriented physiotherapy. The session began with 5 minutes of active-assisted upper and lower limb mobilization exercises (two to three sets of 10-15 repetitions), followed by 5 minutes of joint-specific range-of-motion exercises focusing on restricted or weak joints. Task-oriented upper limb training, including reaching, grasping, and object transfer activities, was conducted for 5 minutes. Sit-to-stand practice (three to four sets of eight to ten repetitions) was undertaken for 5 minutes to improve lower limb strength and functional independence, balance training in sitting and standing positions was performed for 5 minutes, progressing from supported to unsupported tasks, followed by 5 minutes of gait training with progressive reduction of assistance. These sessions were supervised by the rehabilitation team.

By the fourth week of intervention, he showed gradual improvement in proximal limb control, particularly in shoulder elevation and knee extension, allowing him to initiate movements with reduced assistance. His sitting balance improved, and he demonstrated better attention and engagement during the therapy session (Table 2).

At the completion of six weeks, he was able to stand with support using a walker and take a few assisted steps. Distal upper limb control showed mild but meaningful gains, evident in improved grasp strength and smoother release of objects. His MoCA score increased to 20, reflecting better attentional control and recall (Table 2).

At the three-month follow-up, these functional gains were sustained. He was able to walk short distances with assistance and perform basic activities of daily living with partial support. Cognitive performance remained stable, with maintained improvements in attention, language, delayed recall, and orientation domains. Although his motor recovery was incomplete (Table 2), the observed pattern reflected gradual and consistent functional improvement over time. The patient tolerated the intervention well, with no reports of headache, dizziness, or skin irritation.

## Discussion

This case outlines the clinical progression of a 56-year-old male who experienced an ischemic lacunar infarct affecting the right basal ganglia and corona radiata, resulting in left-sided weakness and mild cognitive impairment. Lacunar infarcts, although small, often disrupt significant white matter tracts and cortico-subcortical loops, resulting in motor deficits, psychomotor slowing, and attentional difficulties [12]. The patient's initial neurological status and functional limitations aligned with the anatomical distribution of the lesion.

Anodal tDCS depolarizes neuronal membranes, enhances firing probability, and reinforces synaptic transmission in specific cortical regions. Transcranial direct current stimulation may enhance residual corticospinal output by increasing cortical excitability in the affected primary motor cortex (M1), compensating for impaired subcortical conduction, and facilitating adaptive network reorganization. These mechanisms are consistent with the gradual functional improvements observed in this patient [13-15].

During the six-week intervention, the patient exhibited consistent enhancements in proximal control of both upper and lower limbs, as indicated by the increasing FMA scores. Early changes were primarily observed in proximal limb synergies and trunk control, aligning with existing literature [16] that indicates proximal pathways typically recover sooner due to their extensive cortical representation and redundancy. Improvements in distal hand function occurred at a slower rate, consistent with findings from earlier tDCS studies involving chronic and subacute stroke populations. The observed modest gains in coordination and fine motor tasks indicate the strengthening of the corticospinal and cortico-basal ganglia circuits, which remained structurally intact.

Cognitive enhancements were observed, as indicated by MoCA scores increasing from 16 at baseline to 20 at the three-month follow-up. The improvements were mainly observed in attention, delayed recall, and orientation domains, while visuospatial/executive scores remained unchanged. Rather than indicating substantial cognitive recovery, this pattern suggests stabilization with selective improvement in vulnerable cognitive domains. Given that cognitive impairment following lacunar stroke is often persistent or progressive, maintenance of cognitive performance during the subacute phase may itself be functionally meaningful. These findings are consistent with prior literature suggesting that tDCS may support attentional engagement and cognitive efficiency by modulating fronto-striatal networks commonly affected in small-vessel disease [17,18].

The gradual and slow trajectory of recovery noted in this case reflects typical outcomes in small-vessel ischemic strokes. In contrast to cortical strokes, which may exhibit rapid changes due to early plasticity, lacunar infarcts typically affect deeper motor relay stations, resulting in a more prolonged and gradual recovery process [19]. The patient's transition from wheelchair dependence to assisted ambulation within six weeks, followed by short-distance supported walking, indicates that neuromodulation may have supported functional use of remaining neural pathways.

This case contributes to the accumulating evidence that supports the use of tDCS as an adjunct therapy in motor rehabilitation. Research indicates that the integration of tDCS with task-oriented therapy may facilitate motor recovery, enhance balance, and augment the efficacy of physiotherapy sessions by preparing the brain for learning [20]. This case supports the findings and illustrates the practicality of implementing structured neuromodulation protocols in resource-constrained healthcare environments in India.

While anodal tDCS has been widely explored as an adjunct to physiotherapy in post-stroke rehabilitation, emerging evidence from our recent case study using high-frequency rTMS over the ipsilesional motor cortex suggests complementary neuromodulatory effects on motor and cognitive recovery. Taken together, these findings reinforce the role of targeted non-invasive brain stimulation in modulating disrupted cortico-subcortical networks and supporting functional recovery following lacunar infarction [21].

This case demonstrates strengths such as a detailed longitudinal assessment utilizing standardized tools, consistent therapy scheduling, and clear documentation of motor and cognitive progress at multiple time points. The application of FMA and MoCA facilitated reliable quantification of functional gains, rendering the improvements clinically interpretable [22]. Furthermore, the integration of tDCS in a rural tertiary-care center demonstrates that neuromodulation can be safely implemented outside of highly specialized laboratories.

Nonetheless, it is essential to recognize several limitations. The lack of a control condition or sham stimulation hinders the definitive attribution of improvements exclusively to tDCS. Recovery may have been affected by spontaneous neurological plasticity or solely by physiotherapy [23]. Neurophysiological measures, including motor-evoked potentials and functional imaging, were not collected, which would have enhanced the mechanistic interpretation. The cognitive assessment utilized MoCA exclusively, lacking additional domain-specific evaluations, thereby constraining a comprehensive understanding of cognitive recovery patterns. Ultimately, the findings from this single case should be generalized with caution.

This case underscores the potential advantages of integrating anodal tDCS with structured physiotherapy in the context of subacute lacunar stroke [24]. The ongoing enhancements observed over six weeks, along with the maintained benefits at the three-month follow-up, emphasize the efficacy of neuromodulation as a cost-effective, well-accepted, and scalable intervention appropriate for Indian tertiary-care environments. Future research should investigate larger cohorts, incorporate neurophysiological markers of plasticity, and assess optimal dosing and montage configurations to enhance recovery in small-vessel stroke.

## Conclusions

We treated a patient with a lacunar stroke using a combined program of anodal tDCS and conventional physiotherapy. This case demonstrates that integrating anodal transcranial direct current stimulation with structured physiotherapy may help in enhancing motor recovery and supporting cognitive stability in a patient with an ischemic lacunar infarct of the right basal ganglia and corona radiata. The observed improvements suggest that anodal transcranial direct current stimulation, when used as an adjunct to structured physiotherapy, may be feasible and well tolerated in the subacute phase. Larger controlled studies are required to determine its true efficacy and generalizability.
